# Roll-out of vaccination against COVID-19 pandemic

**DOI:** 10.1186/s40249-021-00902-8

**Published:** 2021-10-18

**Authors:** Xiao-Feng Liang, Guan-Hao He, Wen-Jun Ma, Jian-Peng Xiao

**Affiliations:** 1 School of Medicine, Jinan University, Guangzhou, 510632 China; 2 Guangdong Provincial Institute of Public Health, Guangdong Provincial Center for Disease Control and Prevention, Guangzhou, 511430 China

## Background

With the rebound of coronavirus disease 2019 (COVID-19) epidemic in some countries with high vaccination rate, many concerns on vaccine efficacy have emerged. For example, are the current vaccines ineffective against severe acute respiratory syndrome coronavirus 2 (SARS-COV-2) variants? What’s the status of breakthrough infection? To what extent are non-pharmacological interventions (NPIs) lifted after high vaccination rate? To clarify these questions, we summarized important findings based on literature and Chinese experience.

## Main text

Vaccination is a crucial countermove against infectious diseases by two main mechanisms: (1) prevent infection; (2) reduce infectivity and severity and duration of symptoms of vaccinated patients. Since the outbreak of COVID-19, several vaccines have been developed and proved effective. For instance [1, 2], the efficacy of the vaccines against infection were 90% for the ChAdOx1 nCoV-19 vaccine, 95% for the BNT162b2 mRNA Covid-19 vaccine, 94% for the mRNA-1273 SARS-CoV-2 vaccine, 73–78% for inactivated vaccines (WIVO4 and HBO2), which indicated adenovirus vaccines and mRNA vaccines had higher efficacy than inactivated vaccines. The incidences of severe side effects were 0.7% for adenovirus vaccine, 0.6% for mRNA vaccine, 0.4–0.5% for inactivated vaccine. Vaccine efficacy has also been confirmed by real world data, with 40% lower viral RNA load, 58% lower febrile symptom and 2.3 fewer days of illness duration for vaccinated cases [3]. In addition, during the recent outbreak in Guangzhou, the vaccine efficacy was 69% against infection and 95% against severe symptoms. Therefore, evidence from randomized controlled trials and real world studies verified the effectiveness of COVID-19 vaccines.

Chinese government spares no efforts to promote vaccination. Owing to the high confidence for Chinese government and collectivism of Chinese people, the acceptance rate for COVID-19 vaccine was up to 96.2–100% in China [4]. As of 15th August, 2021, a total of 1862.9 million vaccine doses have been administered in China, with 12.3 million doses daily [5]. The efficient and free vaccination program has contributed to the rapid control of several import-induced outbreaks in China.

The breakthrough infection is another concern. In particular, several SARS-CoV-2 variants have continuously emerged, which may change transmission capacity and virulence. Though cross immunity exists, viral variants can become resistant to the immunity generated by existing vaccines, and the rate of breakthrough infection will inevitably increase [6]. Given that, the vaccination rate for herd immunity should be improved.

Even if the vaccination rate reaches high level, the epidemic could not be controlled without NPIs. For instance, a border city in China, De Hong, had a 96.9% fully vaccination rate [7]. Even so, an import-related outbreak occurred there, and 28 local cases were reported within four days [8], which demonstrated the necessity of NPIs even with high vaccination rate.

## Conclusions

Up to date, vaccination against COVID-19 is effective under the existence of breakthrough infection and viral variants, and the combination of vaccination and NPIs could achieve successful control of COVID-19 in the future.

## Data Availability

Not applicable.

## Abbreviations

COVID-19: Coronavirus disease 2019
NPIs: Non-pharmacological interventions
SARS-COV-2: Severe acute respiratory syndrome coronavirus 2
RCTs: Randomized controlled trials
